# Autocatalytic assembly of a chimeric aminoacyl-RNA synthetase ribozyme

**DOI:** 10.1126/sciadv.adu3693

**Published:** 2025-04-02

**Authors:** Aleksandar Radakovic, Marco Todisco, Anmol Mishra, Jack W. Szostak

**Affiliations:** Howard Hughes Medical Institute, Department of Chemistry, University of Chicago, Chicago IL 60637, USA.

## Abstract

Autocatalytic reactions driving the self-assembly of biological polymers are important for the origin of life, yet few experimental examples of such reactions exist. Here we report an autocatalytic assembly pathway that generates a chimeric, amino acid–bridged aminoacyl-RNA synthetase ribozyme. The noncovalent complex of ribozyme fragments initiates low-level aminoacylation of one of the fragments, which, after loop-closing ligation, generates a highly active covalently linked chimeric ribozyme. The generation of this ribozyme is increasingly efficient over time due to the autocatalytic assembly cycle that sustains the ribozyme over indefinite cycles of serial dilution. Because of its trans activity, this ribozyme also assembles ribozymes distinct from itself, such as the hammerhead, suggesting that RNA aminoacylation, coupled with nonenzymatic ligation, could have facilitated the emergence and propagation of ribozymes.

## Teaser

An RNA-based catalyst is shown to replicate itself indefinitely by incorporating amino acids in its backbone.

## INTRODUCTION

Reactions in which the product catalyzes its own formation are referred to as autocatalytic (*1*–*3*). Drawing inspiration from simple, autocatalytic reactions involving small molecules, more complex autocatalytic schemes that produce enzymes that can catalyze their own synthesis, such as an RNA replicase, have been commonly invoked to explain the origin of life (*4*–*14*). A long-standing goal of the origin-of-life field has been to identify and harness autocatalytic replicative systems involving genetic and/or catalytic polymers. Computational simulations suggest that autocatalytic RNA replicators can, under certain conditions, lead to perpetual propagation of genetic material (*15*–*17*). Yet, despite their importance, only a handful of autocatalytic reactions relevant to the evolution of biochemistry have been reported to date.

The earliest examples of biopolymer autocatalysis leveraged modified, palindromic nucleic acids, which generated exact copies of themselves by nonenzymatic templated ligation of two component oligonucleotides (*18*–*20*). Autocatalysis in these systems was severely inhibited by the strong binding of the ligated product to the template (i.e., another copy of itself). Relying on ribozyme-mediated templated ligation, a ligase ribozyme was engineered to self-assemble autocatalytically by ligating its own component oligonucleotides (*21*). The inherent product inhibition in this system was initially overcome by minimizing the base pairing between the template and the substrate. However, true exponential autocatalysis was achieved after engineering the ligase ribozyme to assemble in a cross-catalytic format with two similar but distinct ligase ribozymes replicating each other (*22*, *23*). More recently, the *Azoarcus* group I intron was shown to be capable of autocatalytic covalent self-assembly via energy-neutral transesterification reactions. Its self-assembly was driven in the forward direction to ~20% full-length product by burying the three key intron recognition base pairs in stem loops after transesterification (*24*). Although the templating feature is generally associated with nucleic acids, it was also used to drive autocatalytic self-assembly of an α-helical peptide that templated a thioester-promoted peptide ligation of its two component peptides (*25*–*28*). While these templated ligation-based autocatalytic systems demonstrated that biopolymer self-replication is possible, they either cannot or have not been shown to assemble or replicate other useful biopolymers. Other autocatalytic reactions, such as formose reaction networks (*29*–*31*), self-reproducing giant vesicles (*32*), autocatalytic micelles (*33*–*35*), and peptide-like replicator fibers (*36*–*38*), have been invoked to model the origin of biochemical processes. However, their connection to the evolution of extant biochemistry remains unclear.

**Fig. 1.**
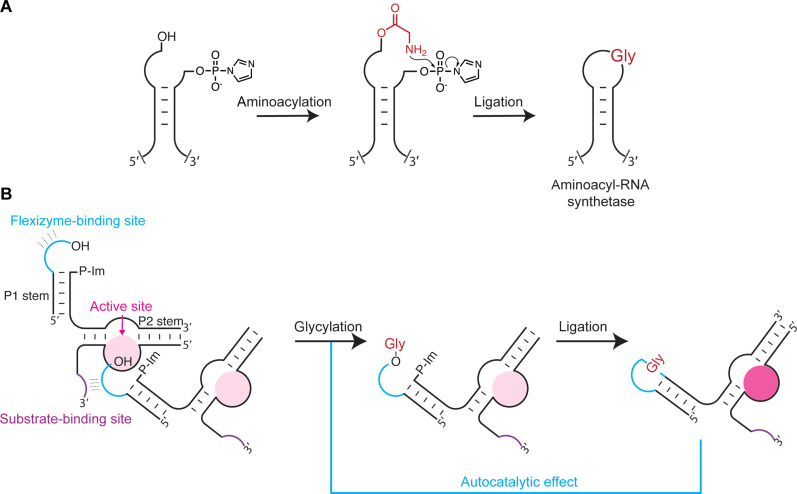
A potential autocatalytic assembly pathway via aminoacylation and loop-closing ligation. (**A**) RNA stems with overhangs become aminoacylated either chemically or enzymatically followed by loop-closing ligation mediated by the amino acid to yield covalently linked chimeric hairpin loops. Ligation of appropriate stem loops leads to a chimeric aminoacyl-RNA synthetase ribozyme [see (*43*)]. (**B**) The proposed autocatalytic assembly cycle using an engineered, chimeric aminoacyl-RNA synthetase ribozyme—the Flexizyme. Glycylation of the cyan overhang followed by loop-closing ligation generates the chimeric ribozyme, which accelerates the initial glycylation leading to autocatalytic self-assembly. The light pink circle represents the weak active site of the noncovalently assembled Flexizyme fragments, while the dark pink circle represents the strong active site of the covalently assembled Flexizyme.

One of the hallmarks of extant life is the mutual dependence of RNA and proteins in ribosomal translation (*39*, *40*), but how this universally conserved machinery emerged remains a mystery (*41*, *42*). We recently discovered that loop-closing ligation of aminoacylated RNA can facilitate the assembly of chimeric ribozymes, thereby linking RNA aminoacylation, an inefficient yet key reaction for the origin of translation, to the efficient construction of RNA-based catalysts (Fig. 1A) (*43*). The loop-closing ligation reaction requires no template, circumventing the formation of catalytically inactive product-template duplexes, which have plagued most RNA-based autocatalytic systems (*19*–*21*, *44*, *45*). Therefore, loop-closing assembly of a chimeric aminoacyl-RNA synthetase ribozyme that could aminoacylate its own component RNAs could in principle proceed indefinitely given sufficient substrates (Fig. 1B). If this chimeric ribozyme could also aminoacylate other RNA substrates, it could participate not only in self-replication but also in the aminoacylation and assembly of other potentially useful chimeric RNAs.

To demonstrate autocatalytic self-assembly, we engineered the Flexizyme aminoacyl-RNA synthetase ribozyme (*46*) to bind and aminoacylate RNA oligonucleotides that, upon loop-closure, yield another copy of the same chimeric, glycine-bridged Flexizyme. The initial aminoacylation that led to the first copy of the chimeric Flexizyme was performed by the noncovalent assembly of the Flexizyme fragments, which retained sufficient aminoacylation activity to initiate the assembly cycle. We measured the kinetic parameters of each individual reaction in the assembly network and built a global kinetic model that accurately predicts the autocatalytic self-assembly cycle. The chimeric Flexizyme was also able to aminoacylate a separate RNA oligonucleotide, which, after nonenzymatic loop-closing ligation, produced an active, chimeric hammerhead ribozyme, a species distinct from the chimeric Flexizyme. This example shows that a self-replicating RNA-based catalyst can assemble other useful RNA-based catalysts. The evolutionary implication of our work is that aminoacylation of RNA, a reaction normally associated with ribosomal translation, could have been at the center of chimeric ribozyme assembly cycles in the earliest cells. These autocatalytic cycles could have operated as primitive hubs of catalyst assembly within the larger, autocatalytic genome replication cycles, which provided the individual RNA oligonucleotide substrates.

## RESULTS

**Fig. 2.**
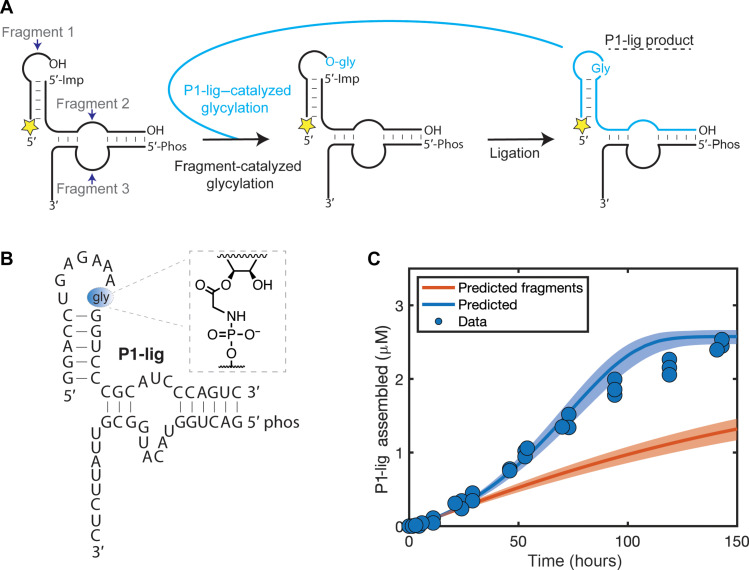
Autocatalytic assembly of the chimeric P1-lig Flexizyme. (**A**) Diagram of the assembly reaction. The fragments self-glycylate via a slow glycylation pathway (black arrow), leading to loop-closing ligation to generate the P1-lig product. The P1-lig product then catalyzes a faster glycylation of the fragments (cyan arrow), leading to more P1-lig. Yellow star: 5′-fluorescein (FAM) label. 5′-Imp represents the 5′-phosphorimidazolide. (**B**) Sequence of the autocatalytic P1-lig Flexizyme, including the close-up of the covalent glycine bridge. (**C**) Assembly of P1-lig over time with 5 μM 5′-FAM–labeled fragment 1, 5 μM 5′-phosphorimidazolide fragment 2, and 0.6 μM fragment 3. The assembly of P1-lig was monitored by acidic denaturing PAGE in three independent replicates by quantifying the percent of fluorescent, ligated product formed (see fig. S4). The filled circles represent the collected data. The blue trace represents the predicted reaction course from the global kinetic model. See fig. S9 for the calculation of error by which the data deviate from the model. The orange trace represents the predicted reaction course if the assembled P1-lig did not enhance the glycylation and deglycylation reactions. Shaded areas represent 95% confidence intervals for predictions of the model.

The Flexizyme contains two stem loops, P1 and P2, that are distant from the catalytic center and that can be manipulated with minimal impact on the activity of the ribozyme (*46*). We modified the stem and loop sequences of the dFx Flexizyme (*46*) so that they become substrates for aminoacylation and loop-closing ligation (*43*). We found that the noncovalently assembled fragments of the modified Flexizyme retained a fraction of the aminoacylation activity of the covalent ribozyme (figs. S1 and S2). Furthermore, the covalent, modified Flexizyme requires only the P1 stem loop to be ligated to display its full catalytic activity (P1-lig hereafter); the P2 stem can remain noncovalently assembled without loss of activity (P2-unlig) (fig. S2). This result alerted us to the possibility of initiating an autocatalytic assembly cycle by simply mixing the Flexizyme fragments and the Flexizyme substrate, the 3,5-dinitrobenzyl ester of glycine (DBE-gly) (Fig. 1B and fig. S3). In this proposed cycle, the low-level activity of the noncovalently assembled fragments generates aminoacylated fragments, which, after loop-closing ligation, result in a highly active covalently linked, chimeric Flexizyme. The chimeric Flexizyme then rapidly aminoacylates more of its component fragments, which ligate to make more of the chimeric Flexizyme, thus driving the autocatalytic assembly cycle (Fig. 2, A and B, and fig. S3).

**Fig. 3.**
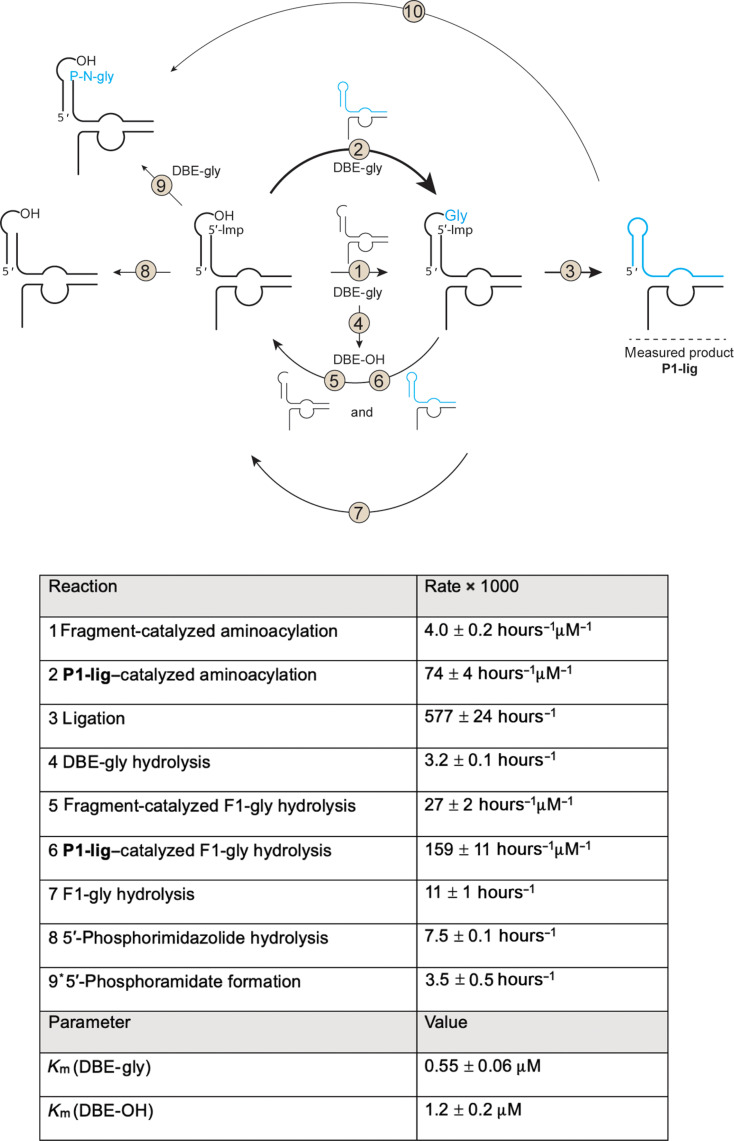
The autocatalytic reaction network. **Top:** The full network of reactions that were characterized in the autocatalytic P1-lig Flexizyme assembly network. The assembled, chimeric P1-lig ribozyme is represented in cyan. The arrow thickness corresponds roughly to the relative reaction rates. **Bottom:** Rates of indicated reactions and Michaelis constant (*K*_m_) values of indicated substrates. Reaction 9* rate was estimated with 20 μM Flexizyme fragments and was not used in the model. Reaction 10 rate was measured in (*43*) and was insignificant for the autocatalytic network.

By monitoring the assembly of the chimeric P1-lig Flexizyme from its fragments, we observed an apparent sigmoidal increase in the yield of the P1-lig ribozyme, consistent with an autocatalytic assembly cycle (Fig. 2C, blue circles). To evaluate whether the sigmoidal kinetics were due to autocatalysis and not the multistep nature of the assembly process, we characterized the kinetics of each reaction in the network independently and built an integrated autocatalytic kinetic model (Fig. 3 and figs. S5 to S8). During our reaction monitoring, we discovered a previously unreported activity of the Flexizyme, namely, the ribozyme-catalyzed hydrolysis of aminoacyl-RNA esters in the presence of DBE-OH, the hydrolysis product of DBE-gly (fig. S5). After accounting for this unusual ribozyme activity, as well as the reactions shown in Fig. 3, our in silico autocatalysis kinetic model predicted similar apparent sigmoidal kinetics that we observed experimentally, with deviations of the model from the data at later time points (Fig. 2C, blue trace, and fig. S9). The trace is not a fit to the data but a prediction of the kinetic model that is based on separately measured individual reaction rates. Our global kinetic model is not based on and is not a fit to the time course of the overall autocatalytic reaction.

**Fig. 4.**
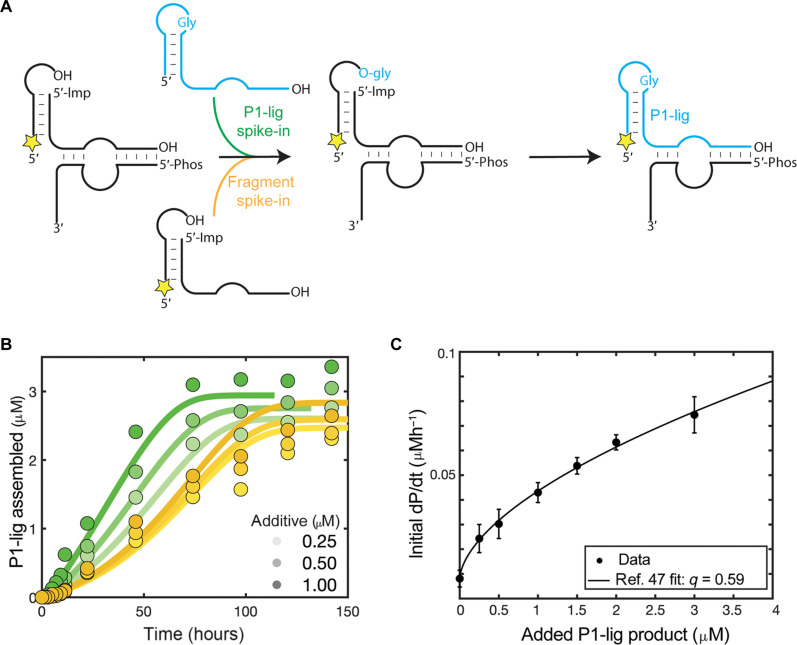
The effect of spiking in the chimeric P1-lig Flexizyme or the fragments on the autocatalytic cycle. (**A**) General scheme of the autocatalytic cycle with either P1-lig (green arrow) or fragment (yellow arrow) spike-in. Yellow star: 5′-FAM label. 5′-Imp represents the 5′-phosphorimidazolide. Note that the spiked in P1-lig product was not 5′-FAM labeled. (**B**) Assembly of P1-lig over time with 5 μM 5′-FAM–labeled fragment 1, 5 μM 5′-phosphorimidazolide fragment 2, and 0.6 μM fragment 3, with the spike-in of either chimeric P1-lig (green) or fragments (yellow) at the additive concentrations of 0.25, 0.5, and 1 μM. The reaction was followed by acidic denaturing PAGE. The filled circles represent the collected data. The traces represent the predicted reaction time course from the global kinetic model. See fig. S9 for the calculation of error by which the data deviate from the model. (**C**) The initial reaction rates (dP/dt; P being the ligated P1-lig chimeric Flexizyme) plotted versus the added chimeric P1-lig product. The data were fit using the fitting procedure in (*47*), with *q* representing the reaction order with respect to the added catalyst, i.e., P1-lig. See fig. S10 for the initial rate data.

With a reasonable kinetic model established, we performed two key tests of whether the self-assembly process was autocatalytic or not. The first test involved performing the reaction in the absence of the catalytic enhancement by the product (i.e., the chimeric P1-lig Flexizyme). We performed this test with our kinetic model in silico because experimentally any changes that abolished the P1-lig activity also abolished the activity of the initial noncovalently assembled fragments. Computationally removing the added aminoacylation activity of the chimeric P1-lig Flexizyme resulted in an attenuated assembly yield and nonsigmoidal kinetics (Fig. 2C, orange trace). The second test for autocatalysis consisted of experimentally spiking in the reaction product at the beginning of the reaction and monitoring the increase in the self-assembly rate (Fig. 4A). Adding the covalently linked chimeric P1-lig product to our fragment system boosted the initial assembly kinetics and the overall reaction yield, an effect that could not be reproduced by simply increasing the concentration of fragments (Fig. 4B, circles). Crucially, the predictions of our kinetic model again were similar to the experimental data, although deviations at later time points persisted (Fig. 4B, traces, and fig. S9).

Adding increasing concentrations of the covalently linked chimeric P1-lig product to the reaction mix allowed us to estimate the reaction order with respect to the catalyst, i.e., the P1-lig product, by plotting the initial reaction rate versus the concentration of the spiked-in catalyst. This procedure is commonly performed to assess the potential of an autocatalytic system for exponential growth. A reaction order of 1 is consistent with the capacity for exponential growth, while values less than 1 indicate an inhibitory mechanism that limits this capacity. We found that the reaction rate increased parabolically with increasing catalyst concentrations (Fig. 4C and fig. S10). Fitting the data with the equation d[P1-lig]/dt = *k*_1_ + *k*_2_[P1-lig_0_]*^q^* [see (*47*)], where *k*_1_ is the rate of the reaction without any catalyst added, *k*_2_ is the autocatalytic rate enhancement, and *q* is the reaction order with respect to the added catalyst, we found the reaction order *q* of 0.59. A reaction order of 0.59 is consistent with inhibition of the autocatalytic enhancement at higher catalyst concentrations, as expected considering our experimental system. In our experiments, fragment 3, which is required for the activity of the P1-lig catalyst, is held constant at 0.6 μM. Because the concentration of the added P1-lig exceeds this value, a parabolic fit to the rate will be observed as fragment 3 becomes saturated with the P1-lig catalyst. Therefore, we can only estimate a lower bound of the reaction order *q* of 0.59, and further careful experiments will be needed to accurately determine the true reaction order with respect to the catalyst. Nevertheless, together, these results are consistent with the proposed autocatalytic assembly cycle shown in Fig. 2A and fig. S3 and inconsistent with a simple multistep assembly process.

**Fig. 5.**
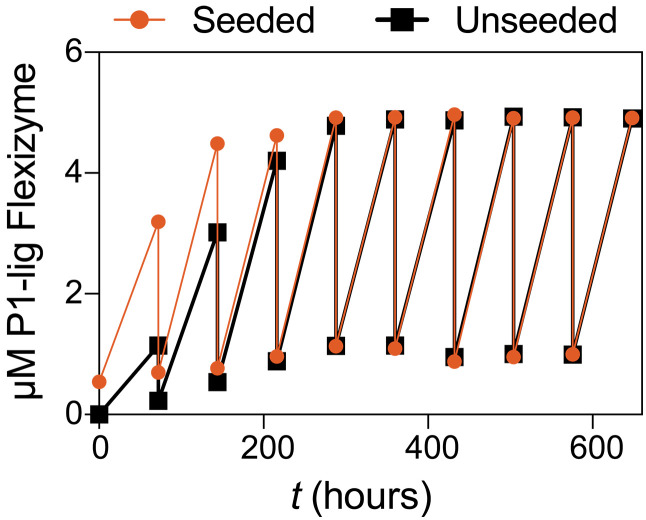
Self-sustained assembly of the chimeric P1-lig Flexizyme. Fourfold serial dilution of the autocatalytic P1-lig Flexizyme assembly reaction into a fresh, uninitiated assembly reaction was performed every 72 hours for 648 hours (black trace). The same procedure was repeated for a reaction that was seeded with 0.5 μM preformed P1-lig Flexizyme (orange trace). Reaction conditions are as follows: 0°C, 100 mM imidazole (pH 8), 5 mM MgCl_2_, 5 μM 5′-FAM–labeled fragment 1, 5 μM 5′-phosphorimidazolide fragment 2, 0.6 μM fragment 3, and 3.38 mM DBE-gly.

An attractive feature of autocatalytic RNA reactions is their potential ability to sustain themselves indefinitely given the addition of fresh substrates, once the reaction is initiated. Templated ligation reactions, with the exception of the cross-replicating and cross-chiral ribozyme ligases (*23*, *48*), cannot sustain themselves indefinitely due to product inhibition. To test whether the autocatalytic P1-lig Flexizyme can sustain itself indefinitely, we allowed the autocatalytic reaction to self-initiate for 72 hours before diluting it fourfold into a freshly prepared uninitiated reaction mixture. We repeated the fourfold dilution every 72 hours for a total of 648 hours. Despite the dilution, the P1-lig Flexizyme assembly reaction accelerated until reaching a plateau after 4 cycles of dilution and assembly (Fig. 5, black trace). With each subsequent dilution, the concentration of the newly synthesized P1-lig Flexizyme increased until it matched the concentration present before dilution. In parallel, we also performed the same serial dilution experiment with a reaction that was seeded with 0.5 μM the preformed P1-lig Flexizyme at the outset. This reaction reached steady-state kinetics after only one dilution, but its steady-state yield was the same as that of the unseeded reaction (Fig. 5, orange trace), suggesting that the advantage of seeding the assembly reaction with the preformed P1-lig Flexizyme becomes erased relatively quickly. Two previous examples of self-sustained autocatalytic RNA assembly used cross-replicating or cross-chiral ligase ribozymes. In both cases, the uncatalyzed background reaction is extremely slow, so that spontaneous self-initiation does not occur and the autocatalytic effect of seeding is maximized. However, this leaves open the question of how, in nature, such a ribozyme could emerge in the first place. In the alternative case of a high spontaneous uncatalyzed reaction rate, the added benefit of autocatalysis would be minimal. Our results are consistent with an intermediate scenario that is relevant for the origins of life, where ribozyme assembly not only self-initiates but also benefits from a relatively strong autocatalytic effect.

**Fig. 6.**
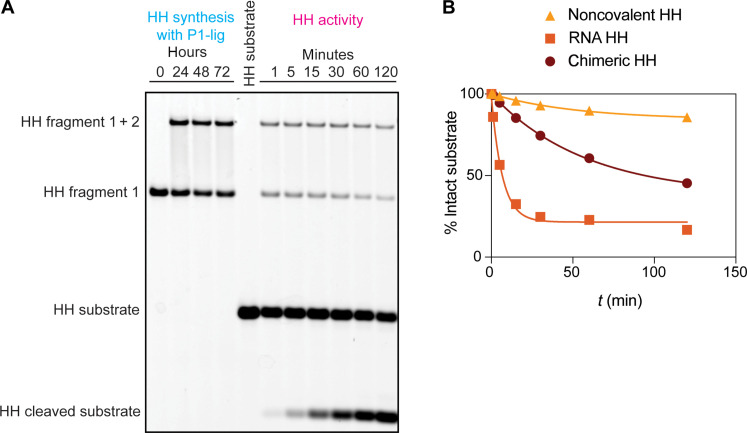
Assembly of an active, chimeric hammerhead (HH) by the chimeric P1-lig Flexizyme. (**A**) Denaturing PAGE of the P1-lig-catalyzed chimeric hammerhead assembly and activity. Conditions for HH synthesis are as follows: 5 mM MgCl_2_, 100 mM imidazole (pH 8), 2 μM chimeric P1-lig Flexizyme, 3.38 mM DBE-gly, 5 μM 5′-FAM–labeled HH fragment 1, and 5 μM HH 5′-phosphorimidazolide fragment 2. Conditions for HH activity are as follows: 37°C, 5 mM MgCl_2_, 100 mM Hepes (pH 7), and 0.2 μM HH substrate. The assembly reaction was diluted 100-fold into the HH activity buffer to initiate the reaction. (**B**) Time course of substrate cleavage by the noncovalently assembled hammerhead, the full-length all-RNA hammerhead, and the chimeric hammerhead. The lines are not fits but are intended to guide the eye. See also fig. S11.

Because the chimeric Flexizyme aminoacylates RNA substrates in trans, it could potentially also facilitate the assembly of other chimeric ribozymes with unrelated functions. To test this possibility, we incubated the chimeric P1-lig Flexizyme with two RNA fragments derived from the hammerhead ribozyme (fig. S11A). We were gratified to observe rapid assembly of the chimeric hammerhead in a reaction that was dependent on the presence of the chimeric Flexizyme (Fig. 6A and fig. S11B). To test the activity of the newly assembled chimeric hammerhead, we diluted the assembly reaction without any purification into the hammerhead reaction buffer containing its substrate. The chimeric hammerhead displayed robust substrate cleavage activity that was substantially higher than the activity of the two noncovalently assembled hammerhead fragments but lower than the full-length, all-RNA hammerhead (Fig. 6B and fig. S11B). This result demonstrates that the autocatalytic P1-lig Flexizyme can not only assemble itself but can also assemble other catalytic chimeric RNAs.

## DISCUSSION

Our work demonstrates that the autocatalytic self-assembly of ribozymes can be driven by aminoacylation followed by loop-closing ligation. Most previously reported autocatalytic ribozymes rely on sequence-defined templated ligation to drive assembly, which eventually leads to the accumulation of nonproductive base-paired duplexes, such that self-assembly becomes off-rate limited. We circumvent this limitation by using aminoacylation coupled with loop-closing ligation, a process that buries the aminoacylated RNA substrate in a highly structured product stem loop (*43*). The formation of the structured loop prevents ribozyme-substrate reassociation, thus avoiding product inhibition of the catalyst. This nontemplated nature of loop-closing ligation allowed us to demonstrate uninhibited, self-sustained assembly of the chimeric glycine-bridged Flexizyme, which can proceed indefinitely over cycles of serial dilution into fresh substrates. Autocatalytic self-assembly of the *Azoarcus* group I intron from its fragments likely also avoids product inhibition by structurally burying the nucleotides involved in base pairing with the catalyst after the ligation step is completed (*24*). A more recent example of uninhibited autocatalytic RNA assembly features two cross-chiral ligases that were evolved in vitro to facilitate multiturnover ligation reactions, thus avoiding the product-catalyst inhibition and sustaining each other indefinitely (*48*). Our autocatalytic chimeric Flexizyme has the added advantage that, in addition to being able to self-assemble, it can also assist in the assembly of other chimeric RNA stem loops if they contain appropriate recognition overhangs. We demonstrate this advantage by using the self-assembled chimeric Flexizyme to assemble a chimeric hammerhead ribozyme through the same process of aminoacylation followed by loop-closing ligation.

We have developed a kinetic model of the chimeric Flexizyme assembly reaction network that relatively accurately predicts the self-assembly process over a range of RNA concentrations. We expect that further optimization of this kinetic model will allow for exploration of the potential for autocatalytic self-assembly under more complex and realistic conditions, for example, in the presence of complex mixtures of RNA oligonucleotides. Incorporating the binding and inhibition parameters of the additional oligonucleotides expected to be present in a self-replicating protocell could lead to more realistic reaction networks directly relevant to the origin of life (*26*, *49*, *50*). We have recently proposed an autocatalytic RNA replication model that could supply the oligonucleotide components that, when combined with the spontaneously initiated autocatalytic self-assembly reported here, could explain how complex ribozymes may have emerged from pools of oligonucleotides (*51*). We are now exploring this exciting possibility. We additionally suggest that the autocatalytic assembly of an aminoacyl-RNA synthetase could facilitate the emergence of diverse ribozymes including ribozymes that enhance RNA replication. Generation of multiple useful ribozymes could help to explain how a self-sustaining genotype could encode multiple self-sustaining phenotypes.

We have described a potentially primordial scenario that would have conferred a fitness advantage to RNAs that participated in aminoacylation, either as substrates or catalysts. In our simple reaction scheme, three relatively short oligonucleotides catalyze aminoacylation of one of the component oligonucleotides, which, after loop-closing assembly, generates a much more efficient RNA aminoacylation catalyst. Indefinite propagation of this catalyst, given a supply of fresh substrates, ensures the preservation of the RNA aminoacylation phenotype by coupling it to self-replication of the RNA aminoacylation catalyst. The aminoacyl-RNA synthetase ribozyme is capable of catalyzing the assembly of other chimeric ribozymes that catalyze unrelated reactions. A general catalyst of RNA aminoacylation (such as the Flexizyme) could incorporate amino acids with diverse side chains into chimeric ribozymes, potentially leading to the emergence of catalysts whose activity depended on the identity of the incorporated amino acid. Such a scenario would create a selection pressure to place the correct amino acid in the correct RNA sequence context, favoring the evolution of specialized aminoacyl-RNA synthetase ribozymes with RNA and amino acid specificity. This sequence of events could potentially explain how the RNA world generated specific and diverse aminoacyl-RNA substrates and synthetases, the essential ingredients for the evolution of translation, without invoking translation itself as the selection pressure.

## MATERIALS AND METHODS

### General information

All reagents were purchased from Sigma-Aldrich and Thermo Fisher Scientific, unless otherwise noted. Oligonucleotide synthesis reagents were purchased from ChemGenes and Glen Research.

### Oligonucleotide synthesis

All oligonucleotides were synthesized in-house on a K&A H-6 instrument. Coupling times were adjusted according to the manufacturer’s instructions (Glen Research or ChemGenes). Oligonucleotides were cleaved, and the nucleobases were deprotected as recommended by the manufacturer. The 2′-O-[(1,1-dimethylethyl)dimethylsilyl] protecting groups were removed by dissolving the oligonucleotides in 100 μl dimethylsulfoxide and 125 μl triethylamine trihydrofluoride and incubating them at 65°C for 2.5 hours. Following precipitation with 0.1 volumes of 5 M ammonium acetate and 1 ml of isopropanol and one wash with 80% aqueous ethanol (v/v), the oligonucleotides were dissolved in 99% formamide (v/v), 5 mM EDTA and purified by denaturing polyacrylamide gel electrophoresis (PAGE). The desired gel bands were isolated, crushed, and soaked in a solution of 5 mM sodium acetate and 2 mM EDTA for 16 hours. The extracted oligonucleotides were desalted using C18 Sep-Pak cartridges (Waters).

### Oligonucleotide activation

*N*-(3-Dimethylaminopropyl)-*N*′-ethylcarbodiimide hydrochloride (EDC.HCl) was added to a 200-μl solution of 5′-phosphorylated oligonucleotides in imidazole (pH 7) buffer so that the final concentrations were 200 μM oligonucleotide, 100 mM imidazole (pH 7), and 100 mM EDC.HCl. The solution was incubated at room temperature for 2 hours before precipitation with 0.1 volumes of sodium perchlorate–saturated acetone and 1 ml of cold acetone. After two washes with cold 1:1 acetone:diethylether (v/v), the pellet was dried and redissolved in 1 mM imidazole (pH 8) buffer. The activated oligonucleotides were stored at −80°C until use.

### Aminoacylation reactions

1) Reaction with Flexizyme fragments: MgCl_2_ and the DBE-gly were added to a solution containing the Flexizyme fragments in imidazole (pH 8) buffer so that the final concentrations were as follows: 5 mM MgCl_2_, 100 mM imidazole (pH 8), DBE-gly (see figure captions for concentrations), and fragments (see figure captions for concentrations). Note that fragment 1 is the fluorescently labeled Flexizyme substrate that is used to measure the aminoacylation activity.

2) Reaction with the covalently linked Flexizyme P1-lig: MgCl_2_ and the DBE-gly were added to a solution containing the covalently linked Flexizyme P1-lig, fragment 3, the fluorescently labeled fragment 1, and the partial complement of the fragment 1 in imidazole (pH 8) buffer so that the final concentrations were as follows: 5 mM MgCl_2_, 100 mM imidazole (pH 8), DBE-gly (see figure captions for concentrations), 0.5 μM P1-lig Flexizyme, 5 μM fragment 1 substrate, and fragment 3 (see figure captions for concentrations). Note that fragment 1 is the fluorescently labeled Flexizyme substrate that is used to measure the aminoacylation activity.

3) Reaction with the covalently linked Flexizyme P1 + P2-lig: MgCl_2_ and the DBE-gly were added to a solution containing the covalently linked Flexizyme P1 + P2-lig and the fluorescently labeled fragment 1 in imidazole (pH 8) buffer so that the final concentrations were as follows: 5 mM MgCl_2_, 100 mM imidazole (pH 8), DBE-gly (see figure captions for concentrations), 0.5 μM P1 + P2-lig Flexizyme, and 5 μM fragment 1 substrate.

Aminoacylation reactions were incubated at 0°C, with 1-μl aliquots quenched in 4 μl of the acidic quenching buffer [10 mM EDTA (pH 8.0), 1× bromophenol blue, 100 mM sodium acetate (pH 5.0), 150 mM HCl, and 75% (v/v) formamide] before loading 2.5 μl of the quenched reactions into acidic 20% denaturing polyacrylamide gels. The acidic gels were cast using 100 mM sodium acetate (pH 5) instead of 1× tris-borate-EDTA buffer. The acidic PAGE was performed at 4°C at 25 W for 3 hours. The gels were scanned in an Amersham Typhoon (Cytiva) gel imager, and the bands were analyzed using ImageQuant TL software. Aminoacylation percentages were obtained by dividing the intensity of the aminoacylated band by the sum of the intensities of the aminoacylated and nonaminoacylated bands for each lane.

### Chimeric Flexizyme synthesis for aminoacylation and spike-in studies

#### P1 + P2-lig Flexizyme

Fragments corresponding to this ribozyme (see fig. S1) with fragments 2 and 3 activated as 5′-phosphorimidazolides were added to a solution of imidazole (pH 8), MgCl_2_, and DBE-gly. Concentrations in the 500 μl of total volume were 5 μM each fragment, 100 mM imidazole (pH 8), 5 mM MgCl_2_, and 3.38 mM DBE-gly. The reaction was incubated at 0°C for 19 hours, before being concentrated to 50 μl using 0.5 ml of 10,000 molecular weight cut-off (MWCO) Amicon filters. The concentrated reactions were mixed with 50 μl of neat formamide and purified on 16% denaturing PAGE. The desired bands were cut out, crushed, and soaked for 3 hours at 4°C in 5 mM sodium acetate and 2 mM EDTA acidified to pH 5 with 1 M HCl. The extracted RNA was concentrated with 0.5 ml of 10,000 MWCO Amicon filters and desalted using the Zymo RNA Clean and Concentrator kit, according to the manufacturer’s protocol.

#### P1-lig Flexizyme

The assembly reaction was set up as above, except that fragment 3 was not activated as 5′-phosphorimidazolide and its final concentration was 1 μM instead of 5 μM (see fig. S1). The reaction was incubated at 0°C for 96 hours before being purified and desalted as above.

### Aminoacyl-RNA hydrolysis reactions

1) Preparative synthesis of the glycylated fragment 1: A 500-μl reaction containing 100 mM imidazole (pH 8), 5 mM MgCl_2_, 10 μM fragment 1, 1 μM dFx_S7 Flexizyme (table S1), and 3.38 mM DBE-gly was allowed to proceed for 48 hours at 0°C. The reaction was concentrated using 0.5 ml of 3000 MWCO Amicon filters to 50 μl, diluted with 50 μl of formamide, and purified by preparative acidic denaturing PAGE. The aminoacylated fragment 1 band was cut out, crushed, and soaked in 5 mM sodium acetate, 2 mM EDTA adjusted to pH 5 for 3 hours at 4°C. The supernatant was filtered, concentrated with 3000 MWCO Amicon filters to 50 μl and precipitated with 5 μl of 3 M sodium acetate (pH 5) and 1 ml of cold ethanol. Following two washes with 80% (v/v) ethanol, the material was analyzed by analytical acidic PAGE to obtain the aminoacylated fraction.

2) The material prepared above was then subjected to hydrolysis under the same conditions as the aminoacylation reaction without the addition of DBE-gly.

3) The deacylation activity of the Flexizyme was characterized by adding DBE-OH, the hydrolysis product of DBE-gly, to the hydrolysis reaction in varying concentrations.

### Aminoacyl-RNA ligation reaction

A 10-μl reaction containing 100 mM imidazole (pH 8), 5 mM MgCl_2_, 5 μM glycylated fragment 1, 5 μM fragment 2 activated as a 5′-phosphorimidazolide, and 3.38 mM DBE-gly was incubated at 0°C. Aliquots (1 μl) were taken at various time points and quenched with 4 μl of quenching buffer [90% (v/v) formamide, 20 mM EDTA, and 1× bromophenol blue]. The quenched aliquots were analyzed by 20% denaturing PAGE, and the percentage of ligated product was obtained by dividing the intensity of the ligated band by the sum of the intensities of the ligated and unligated bands for each lane.

### DBE-gly hydrolysis reaction

A 500-μl reaction containing 100 mM imidazole (pH 8) (prepared in D_2_O), 5 mM MgCl_2_, and 3.38 mM DBE-gly (prepared in d_6_–dimethyl sulfoxide) was incubated at 0°C. ^1^H nuclear magnetic resonance spectra were taken at the indicated time points. The percentage of DBE-gly was obtained by dividing the integrated singlet signal of the two α protons of DBE-gly by the sum of the integrated α proton signal of DBE-gly and DBE-OH.

### 5′-Phosphorimidazolide hydrolysis reaction

A short model oligonucleotide based on fragment 2 (see table S1) was designed to achieve baseline separation between the 5′-phosphorimidazolide activated oligonucleotide and the hydrolyzed 5′-phosphate oligonucleotide by analytical high-performance liquid chromatography (HPLC). The full-length fragment 2 could not be fully resolved to obtain accurate data for the kinetic model. We estimated the percentage of the initial activation of fragment 2, which we used to constrain the kinetic model of the overall assembly reaction, based on the maximum yield of the aminoacyl-RNA ligation reaction.

The 5′-phosphorimidazolide hydrolysis reaction contained 50 μl of 100 mM imidazole (pH 8), 5 mM MgCl_2_, 3.38 mM DBE-gly, 20 μM fragment 1 treated with sodium periodate to prevent 2′,3′-diol aminoacylation and subsequent ligation, 20 μM model oligonucleotide activated as a 5′-phosphorimidazolide, and 20 μM dFx_S7 (see table S1). Fragment 1 and dFx_S7 were included to control for any acceleration of the hydrolysis reaction by the Flexizyme. During the incubation at 0°C, 5-μl aliquots were taken and analyzed by HPLC using the Atlantis TM T3 column (3 μm, 4.6 mm by 150 mm) at a flow rate of 0.5 ml/min. The following gradient was used: (A) aqueous 50 mM triethylammonium acetate (pH 7.0) and (B) acetonitrile, from 6 to 12% B over 20 min. The percentage of 5′-phosphorimidazolide remaining was determined by dividing the area under the signal for the activated species by the sum of the signals for the activated and hydrolyzed oligonucleotide.

### Sodium periodate oxidation of fragment 1

A 500-μl reaction containing 5 μM fragment 1 and 10 mM sodium periodate was allowed to proceed for 1 hour at 0°C. The reaction was then concentrated using 3000 MWCO Amicon filters to 50 μl, followed by precipitation with 5 μl of sodium acetate (pH 5.5) and 1 ml of cold ethanol. After two 80% (v/v) ethanol washes, the pellet was dissolved in ultrapure water and used without further purification.

### Glycine-5′-phosphoramidate formation reaction

A 100-μl reaction containing 100 mM imidazole (pH 8), 5 mM MgCl_2_, 3.38 mM DBE-gly, and 20 μM 5′-phosphorimidazolide activated fragment 2 was incubated at 0°C. After 72 hours, a 5-μl aliquot was analyzed by analytical HPLC as above, resulting in no new chromatographically resolved signals. Another 5-μl aliquot at the 72-hour time point was analyzed on the Agilent 6540 mass spectrometer. Exact masses corresponding to three species were identified and shown in fig. S7.

### The overall assembly reaction of the chimeric Flexizyme from fragments

The assembly reaction was set up at 0°C in a volume of 10 μl containing 5 μM Flexizyme fragments 1 and 2, with the fragment 2 activated as 5′-phosphorimidazolide, 0.6 μM fragment 3, 100 mM imidazole (pH 8), 5 mM MgCl_2_, and 3.38 mM DBE-gly. The reaction was incubated at 0°C, and 0.7-μl aliquots were quenched at various time points in acidic quenching buffer [10 mM EDTA (pH 8.0), 1× bromophenol blue, 100 mM sodium acetate (pH 5.0), 150 mM HCl, and 75% (v/v) formamide] before loading 2.5 μl of the quenched reactions into acidic 20% denaturing polyacrylamide gels. The gels were run and analyzed as above.

The overall assembly reaction of the chimeric Flexizyme from fragments with the addition of preformed P1-lig Flexizyme or fragments was set up as above, except preformed, unlabeled P1-lig Flexizyme or the equivalent molar amount of fragments was added at 0.25, 0.5, and 1 μM.

### Kinetic modeling of the overall assembly reaction

The complex reaction network studied in this work was modeled with a series of coupled first-order differential equations accounting for the transformation of eight species over time as described by the eight chemical reactions in Fig. 3. Time traces were simulated by integrating the model using Euler’s method. Binding of DBE-Gly and DBE-OH to the fragmented Flexizyme and P1-lig was captured by simple hyperbolic curves for competitive binding updated at every integration time step (*52*). Under the assumption of fast reshuffling of all short oligonucleotides constituting the Flexizymes over the reaction timescale, the concentrations of all possible complexes were updated at every integration time step using a MATLAB implementation of the Thomas Wayne Wall algorithm for the solution of multiple chemical equilibria (*50*, *53*). To parameterize and build the model, a set of experiments specifically designed to be maximally sensitive to different and complementary subsets of parameters was fitted either with simple exponentials or with simulated traces from the aforementioned model using MATLAB nonlinear fit algorithm (nlinfit). Errors on the coefficients reported in this work have been derived from the provided variance-covariance matrix. A full list of fitted parameters and associated SEs is presented in Fig. 3.

For simple hydrolysis reactions of labile species, such as reactions number 4 (DBE-gly hydrolysis to DBE-OH), number 7 (hydrolysis-mediated deacylation of fragment 1), and number 8 (5′-phosphorimidazolide hydrolysis to yield a nonreactive 5′-phosphorylated fragment 2), these datasets were individually fitted with a single exponential model with free amplitude to capture any offset in the initial purity of the compounds [see fig. S6 (B to D)].

Given the complexity of the reaction network, no analytical solution for the concentration of species as a function of time is readily available, and we opted to model it using a series of coupled first-order differential equations, integrated using the Euler’s method to generate time traces. The rates and parameters that could not be estimated independently had to be globally fitted using MATLAB nlinfit using simulated traces on data deriving from multiple experiments specifically designed to be maximally sensitive to different and complementary subsets of parameters (see figs. S5 and S6A). Errors on the coefficients here reported have been directly calculated from the variance-covariance matrix provided by MATLAB.

We started to assemble the model based on our understanding as derived from established literature, so that the formation of P1-lig would be the outcome of the sequential reactions of aminoacylation of F1 and loop-closing of the F1-gly and 5′-phosphorimidazolide F2 complex.

Unexpectedly, we found that aminoacylation experiments show a strong nonmonotonic behavior over time, which we could not explain in terms of hydrolysis of the acylated fragment 1 substrate as independently determined for the hydrolysis-mediated deacylation of F1-gly (*k*_7_). A systematic study of this behavior revealed that the drop in acylated products is dependent on Flexizyme concentration and is also dependent on DBE-OH concentration. We therefore hypothesized that this class of ribozymes effectively catalyzes the deacylation of F1-gly using DBE-OH, which accumulates over time due to DBE-gly hydrolysis. We confirmed that the Flexizyme has this secondary catalytic activity, which has not been previously reported.

Measured apparent reaction rates for catalyzed aminoacylation (*k*_1_^*^ and *k*_2_^*^) and catalyzed deacylation (*k*_5_^*^ and *k*_6_^*^) were found to be dependent on DBE-gly and DBE-OH concentrations, respectively. To capture this behavior in our model, the rates for these chemical reactions were updated at every integration step adapting to the relative fraction of Flexizyme bound to either DBE-gly or DBE-OH, so that the effective Flexizyme-catalyzed acylation rates would be

$$k_{1}^{*}={k_{1}}^{*}f_{\text{DBE}-\text{gly}}$$

$$k_{2}^{*}={k_{2}}^{*}f_{\text{DBE}-\text{gly}}$$

and the effective Flexizyme-catalyzed deacylation rates would be

$$k_{5}^{*}={k_{5}}^{*}f_{\text{DBE}-\text{OH}}$$

$$k_{6}^{*}={k_{6}}^{*}f_{\text{DBE}-\text{OH}}$$

The fraction of Flexizyme bound to DBE-gly (*f*_DBE-gly_) and the fraction of ribozyme bound to DBE-OH (*f*_DBE-OH_) were approximated following a simple competitive binding model under the assumption of a common binding site

$$f_{\text{DBE}-\text{gly}}=\frac{\left[\mathrm{DBE}-\text{gly}\right]}{K_{\text{DBE}-\text{gly}}\left(1+\frac{\left[\text{DBE}-\text{OH}\right]}{K_{\text{DBE}-\text{OH}}}\right)+\left[\text{DBE}-\text{gly}\right]}$$

$$f_{\text{DBE}-\text{OH}}=\frac{\left[\text{DBE}-\text{OH}\right]}{K_{\text{DBE}-\text{OH}}\left(1+\frac{\left[\text{DBE}-\text{gly}\right]}{K_{\text{DBE}-\text{gly}}}\right)+\left[\text{DBE}-\text{OH}\right]}$$

In this sense, *k*_1_, *k*_2_, *k*_5_, and *k*_6_ reported in this work can be effectively considered as maximum rates in the limit of Flexizyme-saturated either by DBE-gly (*k*_1_ and *k*_2_) or DBE-OH (*k*_5_ and *k*_6_). The binding constants for DBE-gly and DBE-OH were found to be comparable, supporting the previously established hypothesis that the aromatic moiety is interacting with the Flexizyme binding site and not the amino acid (*46*).

Last, we had to account for the fact that the system is composed of species taking part in multiple equilibria. For example, F1 can be bound to either F2 or F2-im.

Given the large combinatorics raising from having multiple hybridizing RNA strands, we had to use an automated method to update the distribution of species into different complexes. Under the assumption of (i) fast reshuffling of the short oligonucleotides constituting the fragmented Flexizyme over the reaction timescale (*52*) and (ii) strong binding (*K* < < [F1], [F2], [F1-gly], …), the concentrations of all possible complexes could be updated at every integration time step by solving a large system of equations describing all chemical equilibria and mass conservations. Unfortunately, the built-in vpasolve function in MATLAB was too slow to be practically used to solve such a large system and to yield traces usable for globally fitting our large dataset, so we opted for using a custom MATLAB implementation of the very efficient algorithm by Thomas Wayne Wall for the solution of multiple chemical equilibria (*50*, *53*), with a reduction of execution times by two orders of magnitude.

Accounting for all the reactions here described, the overall autocatalytic reaction can be lastly modeled as follows

$$\begin{array}{c} \frac{d\left[F1\right]}{\mathrm{dt}}=-\left[F1\right]\left[R\right]k_{1}^{*}-\left[F1\right]\left[P1:F3\right]k_{2}^{*}+\left[F1-\text{gly}\right]\left[R\right]k_{5}^{*}\\ +\left[F1-\text{gly}\right]\left[P1:F3\right]k_{6}^{*}+\left[F1-\text{gly}\right]k_{7} \end{array}$$

$$\begin{array}{c} \frac{d\left[F1-\text{gly}\right]}{\mathrm{dt}}=\left[F1\right]\left[R\right]k_{1}^{*}+\left[F1\right]\left[P1:F3\right]k_{2}^{*}-\left[F1-\text{gly}\right]\left[R\right]k_{5}^{*}\\ -\left[F1-\text{gly}\right]\left[P1:F3\right]k_{6}^{*}-\left[F1-\text{gly}\right]k_{7}-\left[F1-\text{gly}:F2-\text{im}\right]k_{3} \end{array}$$

$$\frac{d\left[F2\right]}{\mathrm{dt}}=\left[F2-\text{im}\right]k_{8}$$

$$\frac{d\left[F2-\text{im}\right]}{\mathrm{dt}}=-\left[F2-\text{im}\right]k_{8}-\left[F1-\text{gly}:F2-\text{im}\right]k_{3}$$

$$\frac{d\left[P1\right]}{\mathrm{dt}}=\left[F1-\text{gly}:F2-\text{im}\right]k_{3}$$

$$\frac{d\left[\text{DBE}-\text{gly}\right]}{\mathrm{dt}}=-\left[\text{DBE}-\text{gly}\right]k_{4}$$

$$\frac{d\left[\text{DBE}-\text{OH}\right]}{\mathrm{dt}}=\left[\text{DBE}-\text{gly}\right]k_{4}$$

where *R* includes all noncovalent Flexizymes formed by a combination of F1, F1-gly, F2, F2-im and F3, namely, F1:F2:F3, F1-gly:F2:F3, F1:F2-im:F3, and F1-gly:F2-im:F3.

It is important to note that the continuous lines reported in Fig. 2 are predictions and not fits. We found that the model at later times overestimates the reaction yield. We were able to identify a side reaction leading to the formation of a phosphoramidate species from 5′-phosphorimidazolide of fragment 2 and DBE-gly through mass spectrometry (fig. S7). Unfortunately, given that the reaction is dependent both on Flexizyme concentration and DBE-gly, we could not measure it outside of the autocatalytic cycle without using proxy oligonucleotides. To estimate the rate associated with this process, we performed a measurement using 20 μM Flexyzime fragments 1 and 2, where the F1 fragment was periodate-treated (preventing it from being acylated) (fig. S8), leading to an estimate of *k*_9_ and suggesting that at least part of the overestimation could be due to this side reaction.

### Self-sustained assembly of the chimeric P1-lig Flexizyme

#### Unseeded reaction

The assembly reaction was set up at 0°C in a volume of 10 μl containing 5 μM Flexizyme fragments 1 and 2, with fragment 2 activated as a 5′-phosphorimidazolide, 0.6 μM fragment 3, 100 mM imidazole (pH 8), 5 mM MgCl_2_, and 3.38 mM DBE-gly. The reaction was incubated at 0°C for 72 hours after which 2.5 μl of the reaction was mixed with 1 μl of a solution of 6 μM fragment 3 before being added to 6.5 μl of the fresh uninitiated reaction, so that the final volume was 10 μl and final concentrations of the reaction were one quarter of all of the reagents from the previous reaction, 5 μM Flexizyme fragments 1 and 2, with the fragment 2 activated as 5′-phosphorimidazolide, 0.6 μM fragment 3, 100 mM imidazole (pH 8), 5 mM MgCl_2_, and 3.38 mM DBE-gly. The fourfold dilution process was then repeated in the same manner seven more times.

#### Seeded reaction

The reaction was performed as above except the initial reaction contained 5 μM Flexizyme fragments 1 and 2 and 0.5 μM preformed chimeric P1-lig product. To monitor the reaction, 1-μl aliquots were quenched at the initiation, dilution, and completion of the reaction in acidic quenching buffer [10 mM EDTA (pH 8.0), 1× bromophenol blue, 100 mM sodium acetate (pH 5.0), 150 mM HCl, and 75% (v/v) formamide] before loading 2.5 μl of the quenched reactions into 20% denaturing polyacrylamide gels. The gels were run and analyzed as above.

### Chimeric hammerhead assembly and activity

The assembly reaction was set up at 0°C in a volume of 10 μl containing 2 μM unlabeled chimeric Flexizyme P1-lig, 2 μM Flexizyme fragment 3, 5 μM each of hammerhead (HH) fragments 1 and 2, 100 mM imidazole (pH 8), 5 mM MgCl_2_, and 3.38 mM DBE-gly. The reaction was incubated at 0°C, and 0.7-μl aliquots were quenched at 0, 24, 48, and 72 hour time points in 4.3 μl of quenching buffer [90% (v/v) formamide, 20 mM EDTA, and 1× bromophenol blue].

At the 72-hour time point, 1 μl of the assembly reaction was diluted five times with 4 μl of water. After dilution, 1 μl of the diluted assembly reaction was added to 19 μl of the hammerhead activity reaction with the final concentrations being 100 mM Hepes (pH 7), 5 mM MgCl_2_, and 0.2 μM HH substrate. For the RNA HH control reaction, the full-length RNA hammerhead was at 0.05 μM. The hammerhead reaction was then incubated at 37°C, and 2-μl aliquots were quenched at 1-, 5-, 15-, 30-, 60-, and 120-min time points in 8 μl of quenching buffer [90% (v/v) formamide, 20 mM EDTA, and 1× bromophenol blue]. The quenched aliquots were analyzed by 20% denaturing PAGE as described above.
