# Supplementary material for: Autocatalytic assembly of a chimeric aminoacyl-RNA synthetase ribozyme
Source: Sci Adv. 2025 Apr 2;11(14):eadu3693. doi: 10.1126/sciadv.adu3693 (PMC11963975; doi:10.1126/sciadv.adu3693)
Supplement: Supplementary file 1 — Figs. S1 to S11 Table S1 [file sciadv.adu3693_sm.pdf]

Science Advances
AAAS

Supplementary Materials for

Autocatalytic assembly of a chimeric aminoacyl-RNA synthetase ribozyme

Aleksandar Radakovic *et al.*

Corresponding author: Jack W. Szostak, jwszostak@uchicago.edu

Sci. Adv. **11**, eadu3693 (2025)
DOI: 10.1126/sciadv.adu3693

This PDF file includes:

Figs. S1 to S11
Table S1

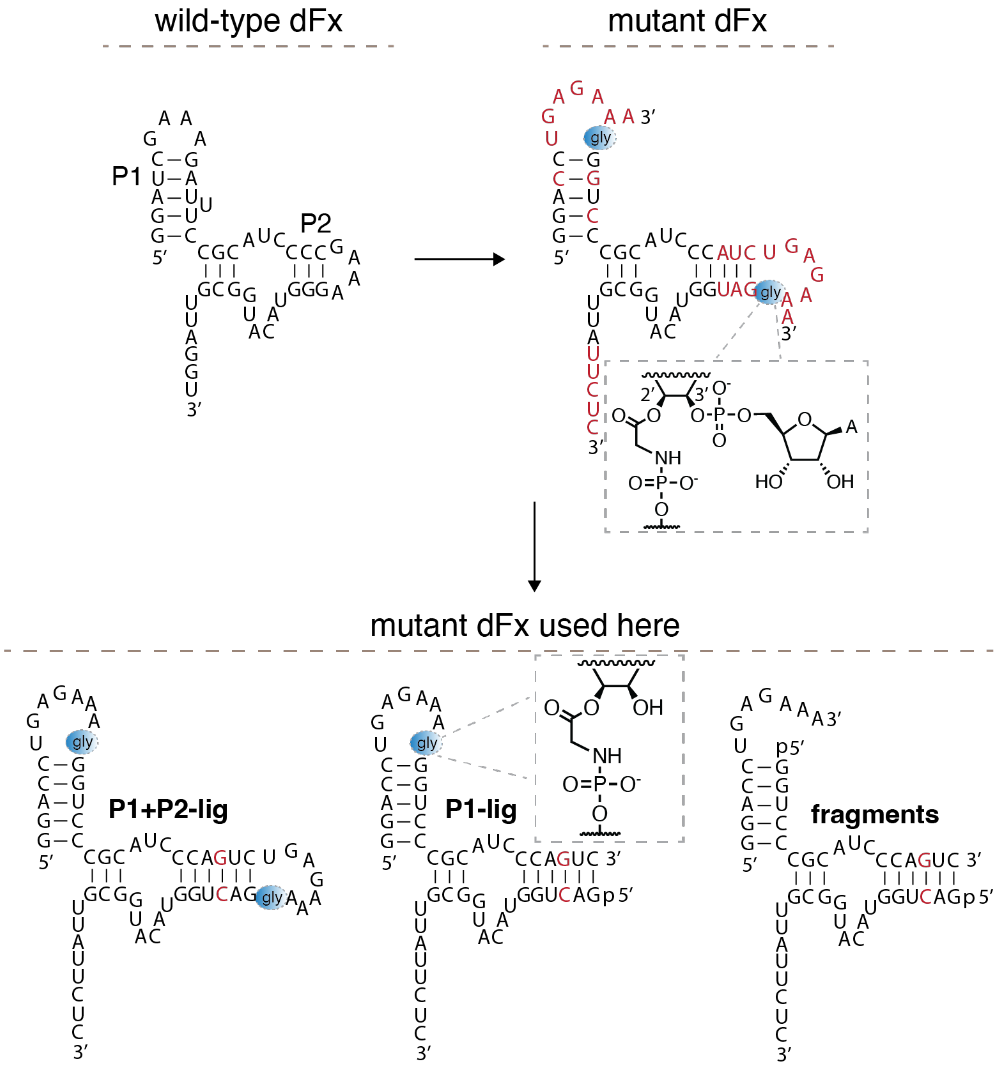

Fig. S1. Alterations of the dFx Flexizyme. Left: The secondary structure of the wild-type dFx Flexizyme. Right: The secondary structure of the dFx with the altered P1 stem, the extended P2 stem, the added 5′-UGAGAAA overhangs on the P1 and P2 stems, and the altered 3′-substrate binding site (modifications in red). The blue circles with "gly" represent the glycine bridges as prepared in ref. 43. Bottom: The secondary structures of the three Flexizyme constructs used in this study. The new modifications are indicated in red. Note that the glycine bridges are between the terminal 3′-nucleotides of the overhangs and 5′-phosphates, due to their Flexizyme-mediated preparation, which exclusively aminoacylates the 2′,3′-diol.

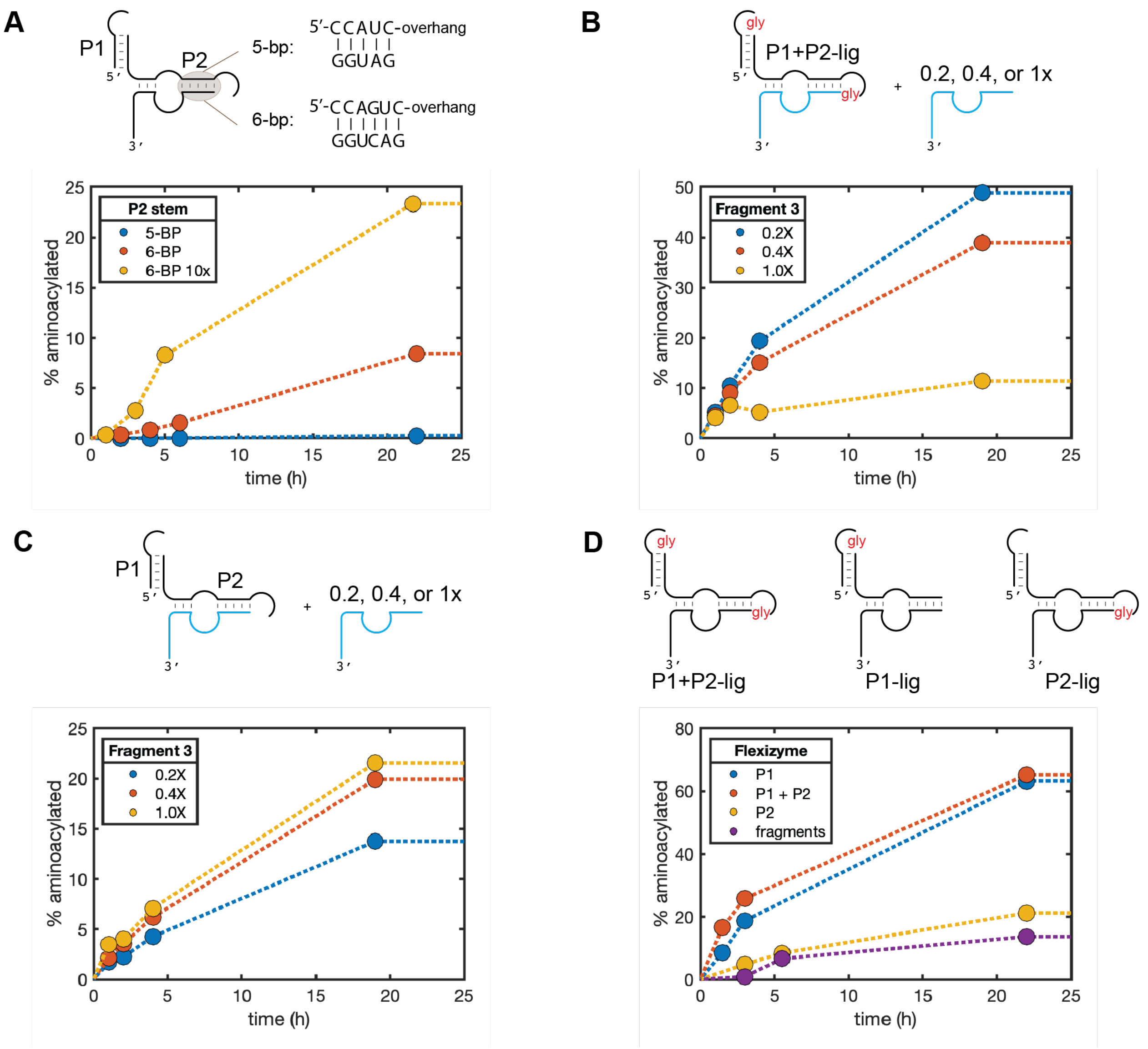

Fig. S2. Aminoacylation optimization with the various fragmented and covalently linked Flexizyme constructs. A: Aminoacylation activity of the Flexizyme fragments where the P2 stem contains 5 or 6 base-pairs was measured. The reactions contained 5 μM of the 5′-FAM fragment 1, 0.5 μM of fragment 2, and 0.5 μM of fragment 3, and 3.38 mM of DBE-gly. The 6-BP 10x condition contained 5 μM each of fragments 2 and 3. **B:** Aminoacylation activity of the fully covalently linked **P1+P2-lig** Flexizyme in the presence of increasing concentration of fragment 3 was measured. The reactions contained 5 μM 5′-FAM labeled fragment 1, 5 μM fragment 2, 1 μM **P1+P2-lig**, 3.38 mM DBE-gly, and either 1 μM, 2 μM or 5 μM of fragment 3 (denoted by 0.2X, 0.4X, and 1X, respectively). The third fragment significantly inhibited the activity of the covalent ribozyme, likely due to occupying the same binding site on the substrate fragment. Dilution of the third fragment relieves this inhibition. **C:** As in **B**, except no **P1+P2-lig** was added. Dilution of the third fragment has minimal impact on the aminoacylation activity of the fragments. **D:** Aminoacylation activity was measured as in **B**, except fragment 3 was kept at the constant 1 μM.

The red “gly” labels represent the glycine bridges. The traces in all four panels connect the data points for ease of visualization and do not correspond to fitted reaction rates.

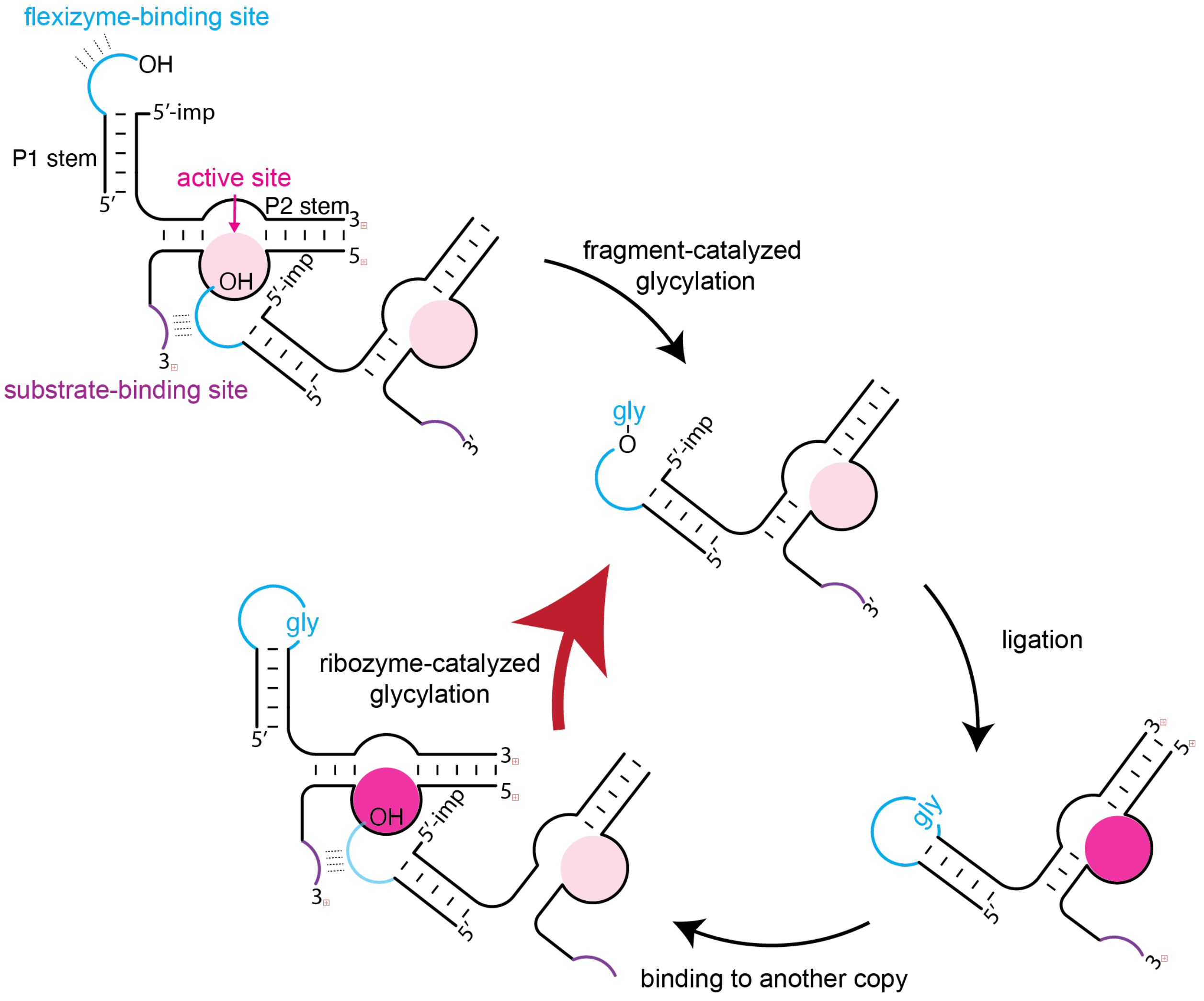

Fig. S3. Autocatalytic assembly of the chimeric P1-lig Flexizyme shown in detail. The chimeric Flexizyme ribozyme assembly according to the simplified scheme shown in Figs. 1B and 2. The overhang in light blue is UGAGAAA-3′, a sequence that can be bound by the purple UUCUC-3′ overhang of the Flexizyme. The active site for aminoacylation is depicted as a pink circle. The low level of activity of the fragments (active site in light pink) is sufficient to aminoacylate another copy of the fragmented ribozyme, which upon loop-closing ligation becomes more active (active site in dark pink) and facilitates its own assembly.

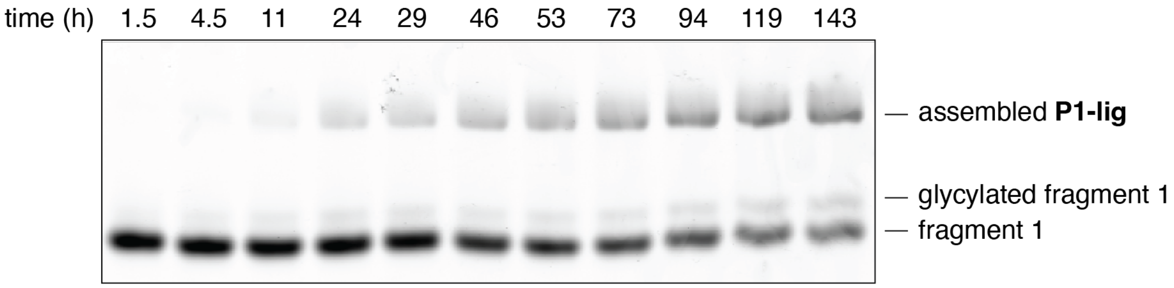

Fig. S4. Representative gel image of the P1-lig Flexizyme assembly reaction. The **P1-lig** Flexizyme assembly reaction was prepared as described in the Methods and Fig. 2C and followed by acidic gel electrophoresis. The bottom-most band represents the 5′-FAM labeled fragment 1. The band just above it represents the glycylated fragment 1. The top-most band represents the assembled **P1-lig** Flexizyme.

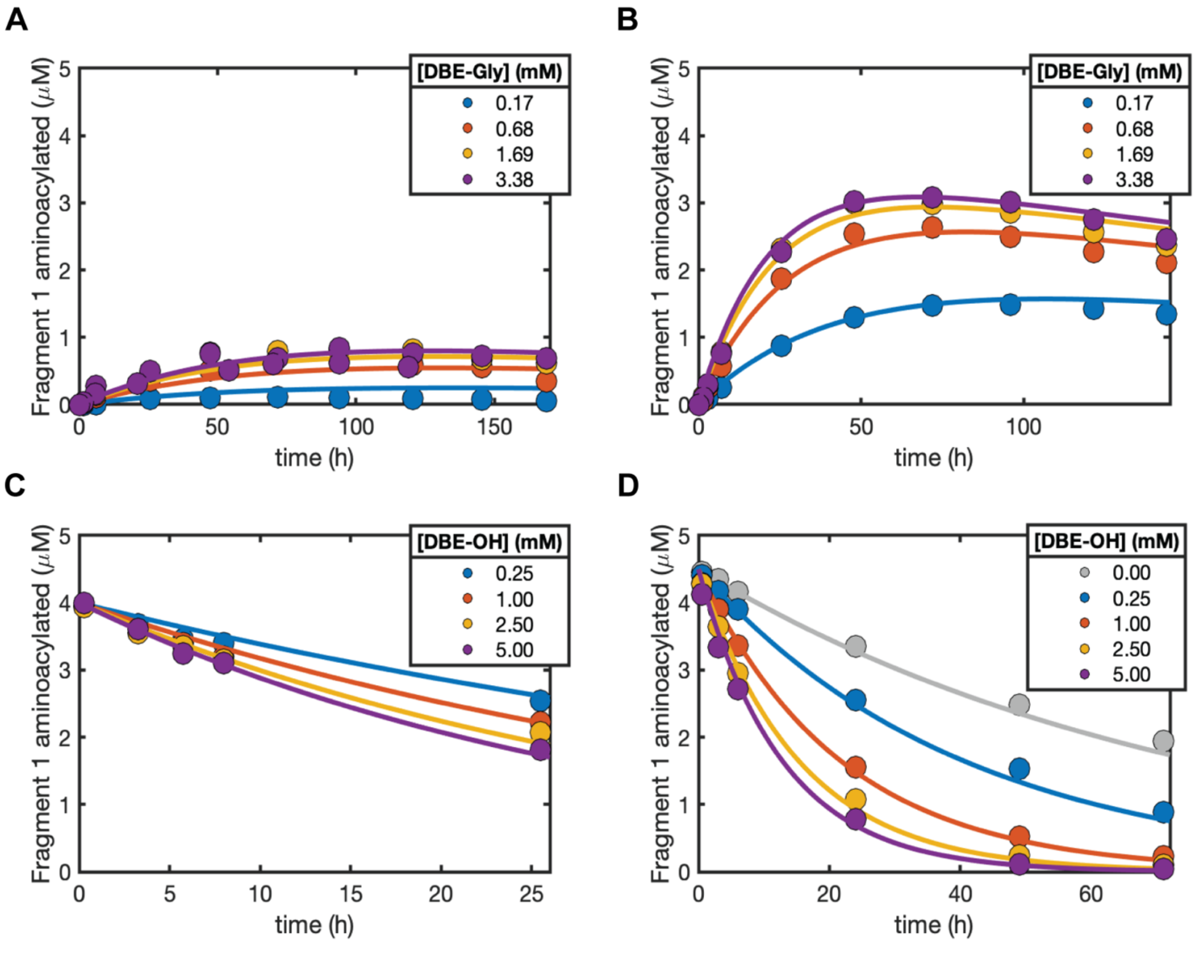

Fig. S5. Aminoacylation and deacylation activities of the fragmented and the P1-lig Flexizyme. A: Aminoacylation activity of the fragments was measured with increasing concentrations of DBE-gly. The reactions contained 5 μM each of the 5′-FAM labeled fragment 1 and 2, and 1 μM of fragment 3. **B:** As in **A**, except the reactions contained 5 μM of the 5′-FAM labeled fragment 1, 5 μM of the model oligonucleotide (Model 2 oligo in Table S1), 0.5 μM fragment 3, and 0.5 μM of the **P1-lig** Flexizyme. **C:** Deacylation activity of the fragments was measured with increasing concentrations of DBE-OH. The reactions were set up as in **A**, except 5 μM of the purified, glycylated and 5′-FAM labeled fragment 1 was used instead of 5 μM of the 5′-FAM labeled fragment 1. **D:** As in **C**, except the reactions contained 5 μM of the glycylated and 5′-FAM labeled fragment 1, 5 μM of the model oligonucleotide (Model 2 oligo in Table S1), 0.5 μM fragment 3, and 0.5 μM of the **P1-lig** Flexizyme. The traces in all four panels corresponded to computationally fitted reaction rates.

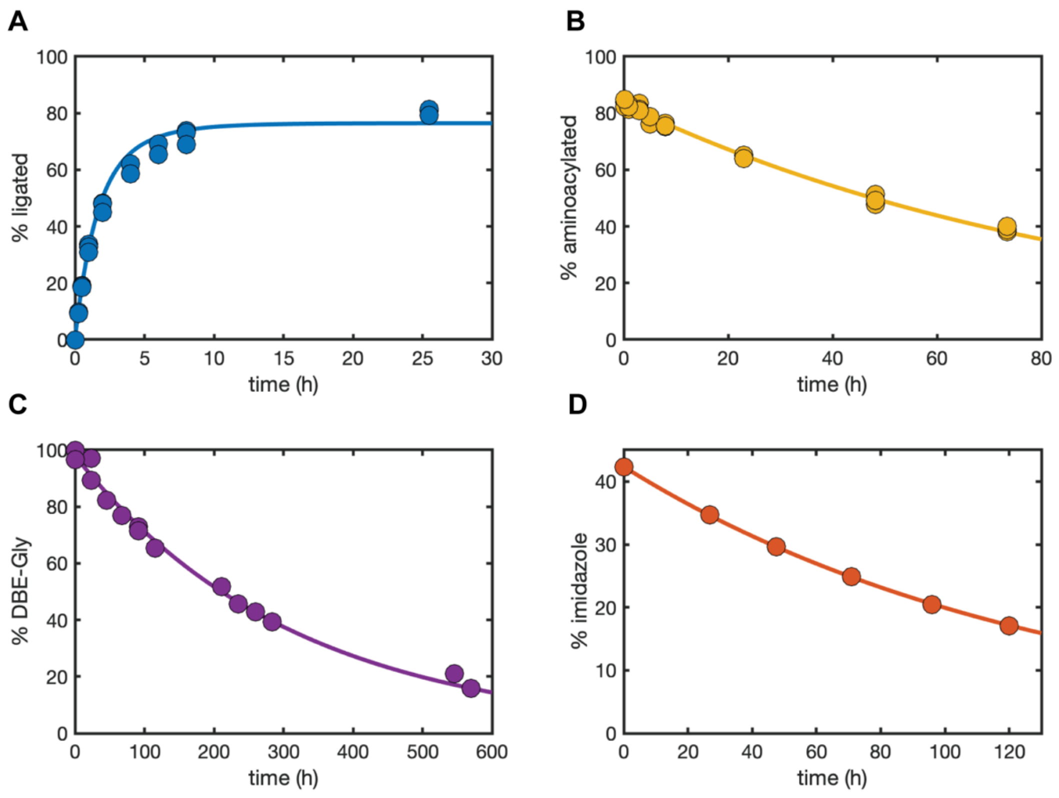

Fig. S6. Kinetics of the loop-closing ligation and hydrolyses in the autocatalytic reaction network. A: The % ligated product was measured by PAGE in a reaction containing 5 µM glycylated and 5′-FAM labeled fragment 1, 5 µM 5′-phosphorimidazolide activated fragment 2, and 3.38 mM DBE-gly. The absence of fragment 3 prevents re-aminoacylation of the hydrolyzed fragment 1 and permits the measurement of the isolated ligation rate. **B:** Hydrolysis of the glycylated and 5′-FAM labeled fragment 1 in the presence of 5 µM fragment 2 and 3.38 mM DBE-gly was measured by acidic PAGE. Re-aminoacylation was prevented as in **A**. **C:** Hydrolysis of DBE-gly to DBE-OH in 100 mM imidazole pH 8 and 5 mM $MgCl_2$ was measured by ^{1}H NMR. **D:** Hydrolysis of the 5′-phosphorimidazolide activated model oligonucleotide (Model 2 oligo in Table S1) was measured by analytical HPLC. The traces in all four panels corresponded to computationally fitted reaction rates.

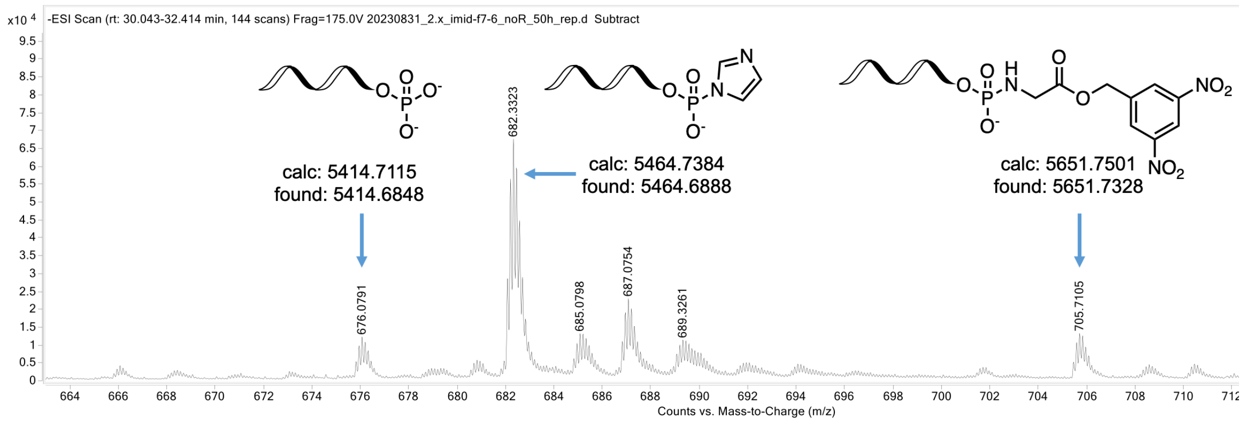

Fig. S7. TOF analysis of the 5′-phosphoramidate formation in the presence of DBE-gly. The 5′-phosphorimidazolide activated fragment 2 was incubated with 3.38 mM DBE-gly in 100 mM imidazole pH 8 and 5 mM $MgCl_2$ for 72 hours. A single signal with ion counts above the noise was found in the total ion chromatogram. Shown is the extracted ion chromatogram that contained the indicated fragment 2 derivatives.

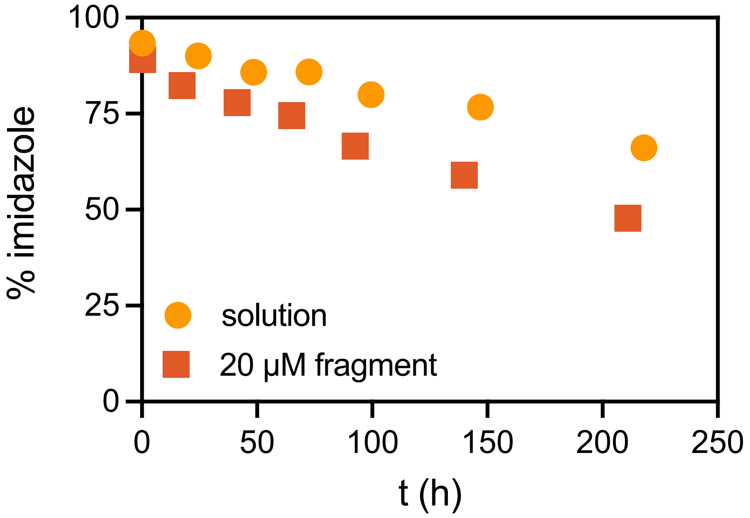

Fig. S8. Flexizyme-catalyzed 5′-phosphoramidate fragment 2 synthesis. The solution reaction contained 100 mM imidazole pH 8, 5 mM $MgCl_2$, 3.38 mM DBE-gly, and 20 µM 5′-phosphorimidazolide fragment 2. The 20 µM fragment reaction additionally contained 20 µM of the periodate-treated fragment 1, and 5 µM fragment 3. Due to the chromatographic overlap of the 5′-phosphoramidate and the 5′-phosphate (the hydrolysis product of the 5′-phosphorimidazolide), we monitored the disappearance of the 5′-phosphorimidazolide fragment 2 via analytical HPLC. The 20 µM fragment reaction accelerated the disappearance of the 5′-phosphorimidazolide fragment 2 and we used the difference in the rates between the 20 µM fragment and solution reactions to estimate the rate of 5′-phosphoramidate synthesis.

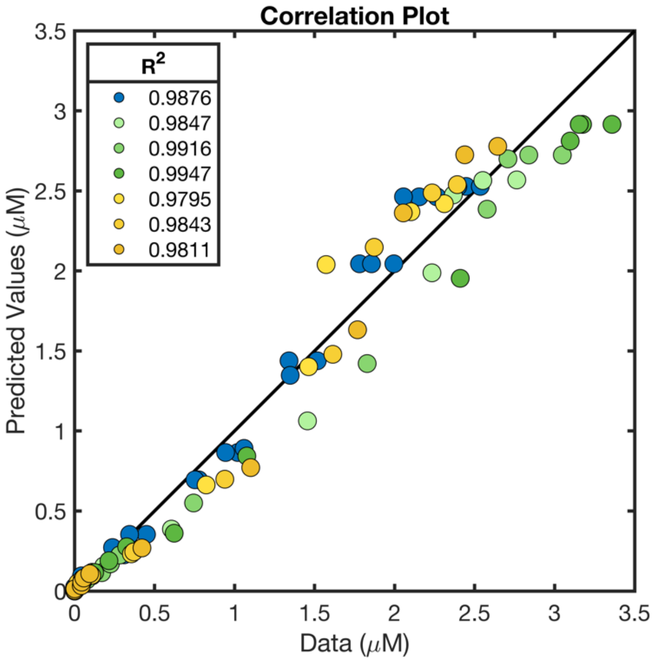

Fig. S9. Correlation plot of the observed data versus the values predicted by the kinetic model. The color of the data points corresponds to the colors used in Fig. 2 (blue) and Fig. 4 (yellows and greens). R^2 was calculated for each data set separately.

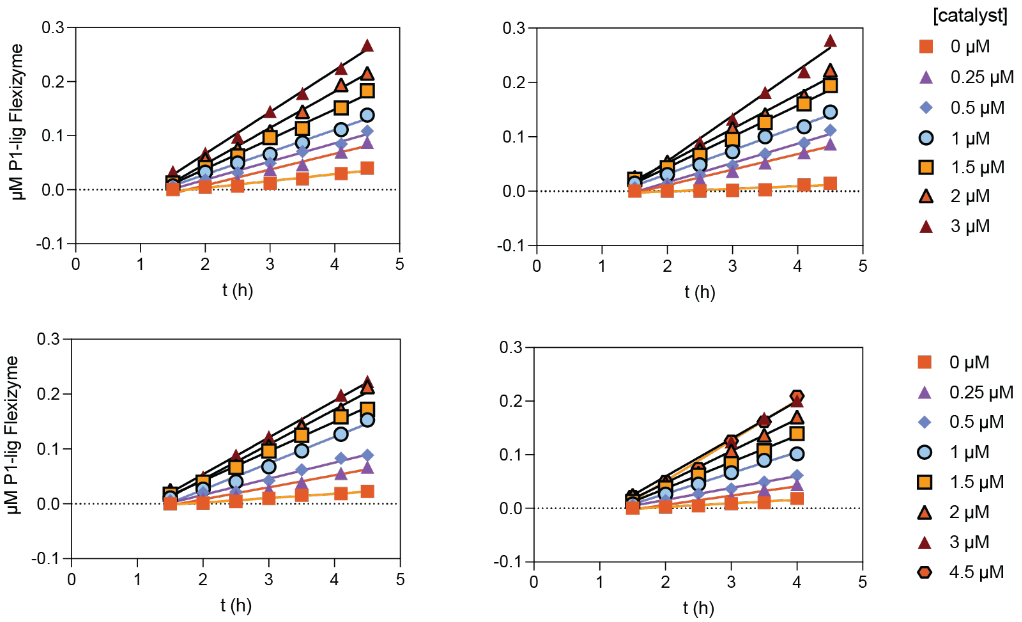

Fig. S10. Spike-in experiment for determining the reaction order with respect to catalyst. Four independent experiments measuring the amount of ligated P1-lig chimeric Flexizyme formed in the initial stages of the autocatalytic assembly reaction with a range of preformed P1-lig Flexizyme spike-in concentrations. The reaction conditions were identical to those used in Fig. 4. Beyond the 4.5-hour timepoint, the product formation is no longer linear. The initial rates of the reaction in µM h^{-1} were obtained from the slope of the linear fit. Note that the data for the 4.5 µM of the added catalyst was not used in Fig. 4C because we did not have sufficient material to perform four independent replicates.

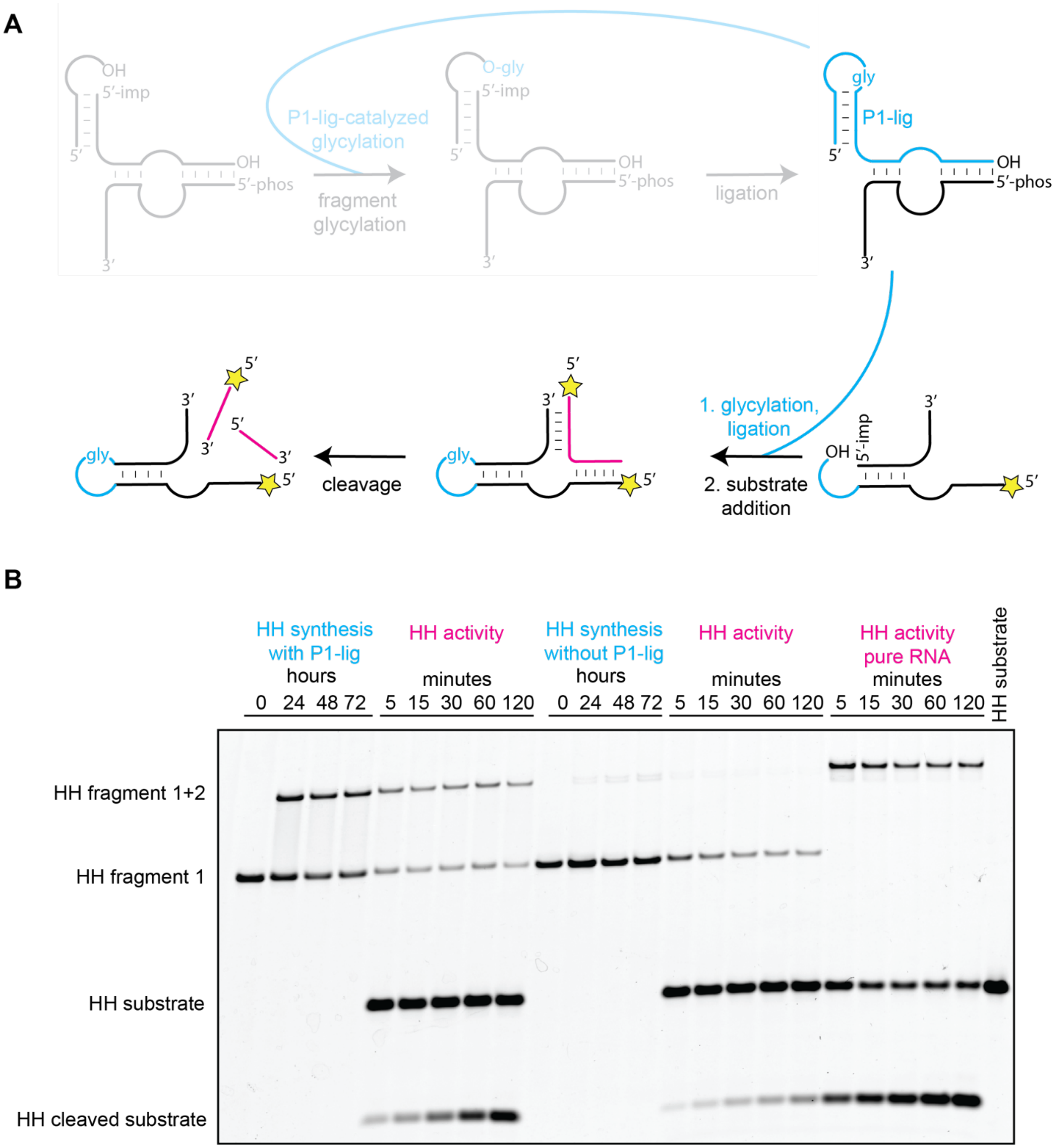

Fig. S11. Chimeric hammerhead synthesis and activity. A: The chimeric P1-lig Flexizyme is first synthesized in the autocatalytic self-assembly cycle (greyed out), then used to aminoacylate the hammerhead fragments, which after ligation form a chimeric stem-loop. The hammerhead substrate is pink. Yellow star represents a 5′-FAM label. Note that the P1-lig ribozyme was not 5′-FAM labeled. **B:** Denaturing PAGE of the hammerhead assembly and activity. Conditions for HH synthesis for the same as in Fig. 5, except in the case of 'HH synthesis without P1-lig' where no P1-lig ribozyme was added. Conditions for HH activity were the same as in Fig. 6.

Table S1. RNA sequences used in this work. Imp represents imidazole activated phosphate.

Name	Sequence
Fragment 1 (F1)	5′-fluorescein-GGACCUGAGAAA
Fragment 2 (F2)	5′-phos/Imp-GGUCCCGCAUCCCAGUC
Fragment 3 (F3)	5′-phos-GACUGGUACAUGGCGUUAUUCUC
Fragment 2_overhang	5′-phos/Imp-GGUCCCGCAUCCCAGUCUGAGAAA
Fragment 2_5BP	5′-phos-GGUCCCGCAUCCCAUCUGAGAAA
Fragment 3_5BP	5′-phos-GAUGGUACAUGGCGUUAUUCUC
**P1+P2-lig**	5′-fluorescein-GGACCUGAGAAA-gly-GGUCCCGCAUCCCAGUCUGAGAAA-gly-GACUGGUACAUGGCGUUAUUCUC
**P1-lig**	5′-fluorescein-GGACCUGAGAAA-gly-GGUCCCGCAUCCCAGUC
**P2-lig**	5′-phos-GGUCCCGCAUCCCAGUCUGAGAAA-gly-GACUGGUACAUGGCGUUAUUCUC
Model 2	5′-phos-GGUCC
dFx_S7	5′-fluorescein-GGACCUGAGAAA GGUCCCGCAUCCCAUCUGAGAAA GAUGGUACAUGGCGUUAUUCUC
**P1-lig** unlabeled	5′-OH-GGACCUGAGAAA-gly-GGUCCCGCAUCCCAGUC
HH1	5′-fluorescein-AAGCACACUGAUGAGCCUUGAGAAA
HH2	5′-phos/Imp-AGGCGAAACGAU
HH substrate	5′-fluorescein-AGAUCGUCUGUGC
HH all-RNA	5′-fluorescein-AAGCACACUGAUGAGCCUUGAGAAAGGCGAAACGAU
